# Role of the Antioxidant Activity of Melatonin in Myocardial Ischemia-Reperfusion Injury

**DOI:** 10.3390/antiox11040627

**Published:** 2022-03-25

**Authors:** Jorge Luis Bermudez-Gonzalez, Denya Sanchez-Quintero, Leonardo Proaño-Bernal, Rafael Santana-Apreza, Marco Antonio Jimenez-Chavarria, Jose Antonio Luna-Alvarez-Amezquita, Juan Ignacio Straface, Arantza Marie Perez-Partida, Joaquin Berarducci, Javier Ivan Armenta-Moreno, Karla Joana Garza-Cruz, Nilda Espinola-Zavaleta, Erick Alexanderson-Rosas

**Affiliations:** 1 National Institute of Cardiology Ignacio Chavez, Juan Badiano 1, Belisario Dominguez Secc 16, Tlalpan, Mexico City 14080, Mexico; jorgebermudez26@comunidad.unam.mx (J.L.B.-G.); joseluna@lasallistas.org.mx (J.A.L.-A.-A.); juan.straface@anahuac.mx (J.I.S.); arantza.perezpa@anahuac.mx (A.M.P.-P.); jberarducci96@gmail.com (J.B.); javierivan_am@hotmail.com (J.I.A.-M.); karlagacz@gmail.com (K.J.G.-C.); 2 Faculty of Medicine, National Autonomous University of Mexico, Escolar 411 A, Copilco Universidad, Coyoacan, Mexico City 04360, Mexico; mc20saqd5602@facmed.unam.mx (D.S.-Q.); leoprob.98@comunidad.unam.mx (L.P.-B.); rafasantana1911@gmail.com (R.S.-A.); mc20jicm6557@facmed.unam.mx (M.A.J.-C.)

**Keywords:** melatonin, ischemia-reperfusion, coronary artery disease, circadian rhythms, animal model, myocardial infarction

## Abstract

Ischemia-reperfusion injury is a common problem in the age of interventional cardiology; it is primarily mediated by oxidative stress and reactive agents. Melatonin has antioxidative properties that make its use promising for treating ischemia-reperfusion injury. Multiple experimental studies in murine and porcine models have been performed with good results. Clinical trials have also been conducted but given their heterogeneity, no conclusive results can be made. Melatonin pharmacokinetic properties are not ideal; therefore, many analogs have been proposed with improved characteristics, and some studies have evaluated their efficacy in animal models, but clinical trials are needed to recommend their use. In this review, we expose the results of the most impactful studies regarding melatonin use in ischemia-reperfusion injury.

## 1. Introduction

In this age of increasing techniques and availability of reperfusion treatments, ischemia-reperfusion injury has become an important problem in the interventioal cardiology field [1,2]. Although reperfusion is the treatment of choice for multiple patients, this procedure is not innocuous. It has been established that multiple factors, related mainly to oxidative stress, mitochondrial dysfunction, and cellular death, contribute to myocardial injury following the reperfusion event, leading to myocardial stunning, arrhythmias, increased infarct size, and heart failure. To prevent these situations, therapeutic agents and procedures have been developed, aiming to reduce cellular damage secondary to reperfusion.

Melatonin has an important antioxidant activity and has shown promising results in reducing myocardial reperfusion injury in numerous animal models. Clinical trials assessing its efficacy for ischemia-reperfusion injury have been performed, however, since it is a novel agent, its route of administration and adequate dosage is not well established, leading to heterogeneous outcomes. Pure melatonin has limited pharmacokinetics due to an oral bioavailability of 9 to 33% [3]. In this review, we discuss several experimental and clinical studies evaluating melatonin’s role in myocardial ischemia-reperfusion injury. We also mention melatonin analogs, which possess improved pharmacokinetics and may be helpful in ischemia-reperfusion injury.

## 2. Melatonin and Physiology

Melatonin (N-acetyl-5-methoxytryptamine) is an indoleamine hormone mainly synthesized by the pineal gland and directly released into the bloodstream. It is an ancient molecule whose presence is not restricted to vertebrates, as it is also found in invertebrates, unicellular organisms, and plants, none of which possess a pineal gland [4]. Nevertheless, there is also an “extrapineal melatonin production”; melatonin has been identified in a wide variety of sites, such as the brain, the retina, the lens, and the gastrointestinal and reproductive tract. In non-primate mammals (though with exceptions), it is also produced in the Harderian gland [5].

This suggests that melatonin first appeared when organisms began to use oxygen, and its original role was to neutralize the toxic O_2_ derivatives (free radicals and reactive oxygen species), thus, acting as an antioxidant [6]. Although melatonin’s structure has not changed, its original role as an antioxidant was preserved and complemented by a diversity of other actions because of evolution [6].

### 2.1. Byosynthesis and Secretion

**Figure 1:**
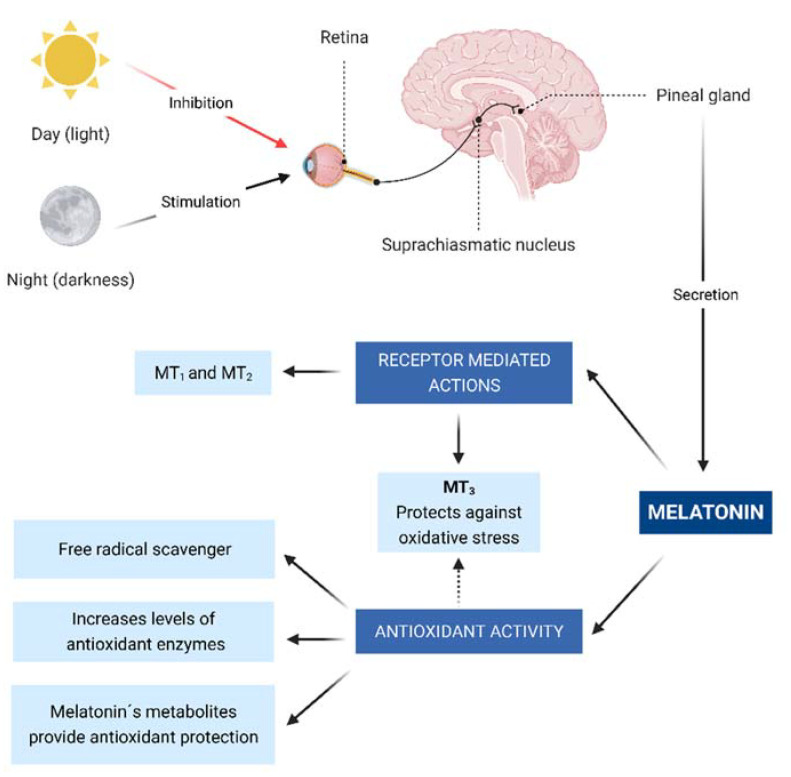
Neural control of pineal melatonin synthesis and its physiological effects via receptors mediated and non-mediated actions (antioxidant activity). Daylight inhibits the pineal production of melatonin while darkness stimulates it. Melatonin acts in receptors MT 1 to 3. MT3 is important due to its antioxidative activities.

Melatonin is secreted in a circadian pattern (Figure 1) and its secretion is activated in low light conditions. Consequently, during nighttime, melatonin is released through a multisynaptic pathway that starts in the suprachiasmatic nucleus and ends in the pineal gland [7].

### 2.2. Receptor-Mediated Actions

Melatonin produces multiple effects once it is released into the bloodstream, some of which are receptor-mediated. In humans, 2 receptors (MT1 and MT2) have been characterized as those that perform these actions. MT1 and MT2, located in most peripheral tissues and the central nervous system, are transmembrane receptors that belong to the family of G protein-coupled receptors, which act through second messengers, including adenylyl cyclase, phospholipase A2, and phospholipase C. These messengers will, in turn, modify the production of cAMP and cGMP, or diacylglycerol and IP3 [8].

In addition, a third cytoplasmic melatonin receptor called MT3 was later characterized as a quinone reductase enzyme whose function is to inhibit the electron transfer of quinones, thus protecting against oxidative stress [9].

MT1 and MT2 are the only receptors in humans that have been found to play a role in the cardiovascular system, being present in cerebral arteries, coronary arteries, systemic arteries, aorta, and cardiac ventricular wall [10,11]. These findings support the idea that melatonin may have a significant impact on a variety of cardiovascular diseases [12].

### 2.3. Antioxidant Activity and Anti-Inflammatory Effect

In addition to receptor-mediated actions, melatonin also has numerous antioxidant effects.

The first of these effects is mediated via the MT3 receptor, which acts as a quinone reductase, inhibiting electron transfer from quinones and reducing oxidative stress [7,13].

Melatonin also donates electrons, decreasing reactive oxygen (ROS) and nitrogen species (RNS), such as nitric oxide (NO), superoxide anion radical (O^2−^), and hydroxyl radical (OH), etc. Therefore, melatonin not only acts through receptors but also acts directly as a free radical scavenger [14,15].

In addition to these two main mechanisms, melatonin is also responsible for increasing the levels of antioxidant enzymes via MT1 and MT2. These enzymes include superoxide dismutase, glutathione peroxidase, glutathione reductase, and glucose-6-phosphate dehydrogenase, thus decreasing molecular damage [16].

On the other hand, melatonin radical free scavenging activity also extends to its metabolites, which are also potent electron donors and provide antioxidant protection by the same mechanisms that have been previously described. As a result, this cascade of the first, second, and third-generation metabolites of melatonin allows this molecule to detoxify up to 10 oxidizing molecules [17].

Melatonin, distinct from certain antioxidants such as glutathione, vitamin C, and E, does not undergo complete redox cycles; rather, it is only reduced and does not release electrons, preventing the oxidation of cellular compounds by reducing reactive oxygen species [18].

Another major antioxidant function of melatonin is that of reducing nitrosative damage by neutralizing toxic nitrogen compounds such as nitric oxide and peroxynitrite anion, which generate nitrosative damage. Melatonin is also responsible for its suppressing effect on the enzyme nitric oxide synthase, which increases oxidative stress. Both actions regarding nitric oxide metabolism are important in the context of ischemia-reperfusion injury [14].

Due to its lipophilic properties, melatonin can easily cross membranes. This is especially important since melatonin, by easily reaching the mitochondria, allows it to maintain an adequate function of this organelle by exerting antioxidant properties, reducing oxidative stress, and reducing the possibility of events such as apoptosis and cell death [14].

Regarding its anti-inflammatory effects, melatonin can stimulate the secretion of anti-inflammatory mediators (IL-10) while inhibiting that of pro-inflammatory cytokines such as TNF-α, IL-6, and IL-1β, which are responsible for tissue injury [19]. Melatonin is also responsible for inhibiting the TLR4 reaction pathway, which is one of the factors responsible for tissue damage after myocardial infarction [20].

Dyslipidemia and obesity are important factors for the development of coronary heart disease. Melatonin has been shown to have beneficial effects on dyslipidemia by decreasing total cholesterol, LDL cholesterol, ox-LDL, and lipid peroxidation. It has also been described that melatonin has anti-obesity effects. Both actions protect against the development of myocardial infarction [21].

Melatonin also plays an important role in the development of atherosclerosis through the regulation of the MAPK pathway and suppression of the activity of myosin light-chain kinase (MLCK), therefore decreasing endothelial dysfunction and the formation of atheromatous plaques [22].

## 3. Overview of Ischemia-Reperfusion Injury

**Figure 2:**
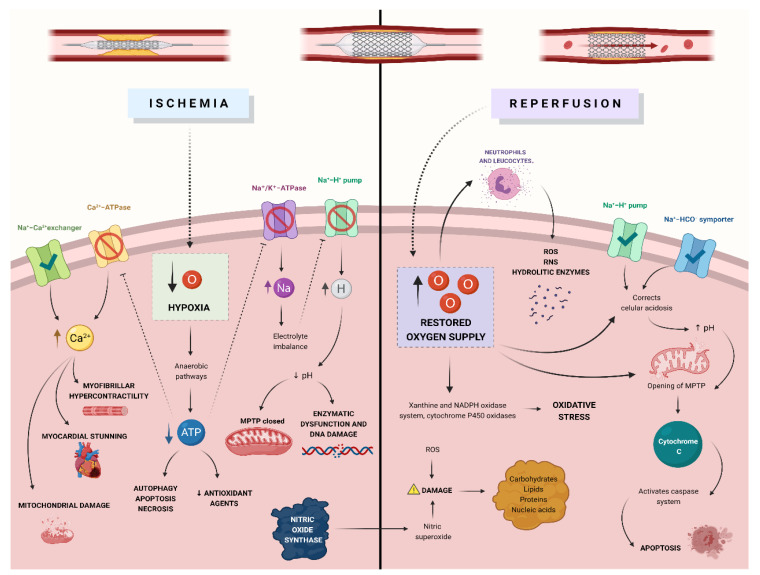
Pathophysiology of ischemia-reperfusion injury. The mechanisms through which myocardial tissue is damaged after the restoration of blood flow following a period of ischemia include oxidative stress, ion accumulation due to the dysfunction of cellular pumps, nitric oxide species that produce damage, mitochondrial dysfunction through the opening of MPTP and activation of cellular death pathways.

**Figure 3:**
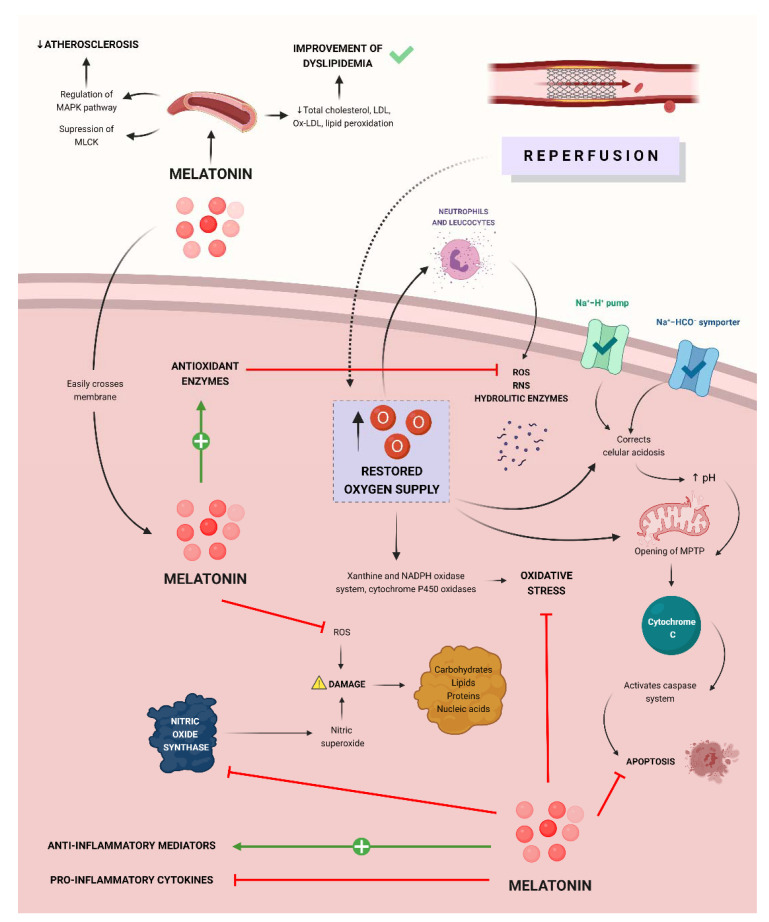
Mechanisms through which melatonin exerts myocardial protection in MI/R injury. Melatonin is capable of inhibiting the nitric oxide synthase, pro-inflammatory cytokines, ROS, and cellular pathways conducting to apoptosis. It is also responsible for enhancing anti-inflammatory mediators and antioxidant enzymes. Extracellularly, melatonin decreases atherosclerosis and improves dyslipidemias, which reduce the risk of ischemic episodes.

In 1960, Jennings described ischemia-reperfusion injury, a condition where myocardial tissue could be damaged after the restoration of blood flow [23]. Ischemia-reperfusion injury has been linked to clinical problems such as myocardial stunning and acute heart failure [24]; given its clinical relevance and its potential as a therapeutic target, it is of vital importance to know the multiple physiopathological mechanisms involved in this type of injury (Figure 2 and Figure 3).

### 3.1. Oxidative Stress

During the obstruction of arterial blood flow, cells experience hypoxia, shifting cell metabolism from aerobic to anaerobic pathways [24]. The result is a reduction in the concentrations of ATP and antioxidative agents [24]. When perfusion is restored in an ischemic region, the new supply of oxygen to cells that have impaired antioxidant mechanisms produces an increase in reactive oxygen species [25]. 

Mechanisms that produce oxidative stress can be divided into those that do not need enzymes and those that do; the latter (xanthine oxidase system, NADPH oxidase system, cytochrome P450 oxidases) have the most impact in ischemia-reperfusion injury [24,25]. Xanthine oxidoreductase is an enzymatic complex that can create reactive oxygen species in situations where energy input plummets (e.g., ischemia) [26]. During reperfusion, xanthine oxidase uses oxygen to create xanthine and uric acid, and this process releases superoxide and hydrogen peroxide [26]. 

NADPH oxidases between 1 and 5 are involved in the production of reactive oxygen species during ischemic-reperfusion injury [26]. These enzymes can be induced and upregulated by sterile inflammation, producing large quantities of superoxide in the process [24,26]. Cytochrome P450 oxidases are also capable of producing eicosanoid derivatives and reactive oxygen species during ischemic-reperfusion injury [26].

### 3.2. Ion Accumulation

Ischemia produces dysfunction in numerous cellular pumps [22]. Dysfunction in the Na⁺/K⁺-ATPase pump produces sodium ions accumulation inside the cell and potassium outside the cell, this electrolyte imbalance induces dysfunction in the Na^+^-H^+^ pumps [22]. The retention of hydrogen ions decreases intracellular pH, which then causes enzymatic dysfunction and DNA damage [22]. During reperfusion, cellular acidosis is reverted by the activation of the Na^+^-H^+^ pump and the Na^+^-HCO^−^ symporter [24]. The rapid correction of pH during reperfusion after an episode of ischemia produces cellular damage by the opening of mitochondrial permeability transition pores, this mechanism is known as the pH paradox [24].

Ca^2+^-ATPases pumps at the endoplasmic reticulum become dysfunctional during ischemia due to low concentrations of ATP, this alteration limits calcium reuptake by the sarcoplasmic reticulum [22,24]. Additionally, higher intracellular sodium concentrations result in activating the Na^+^-Ca^2+^ exchanger. These alterations increase the intracellular concentration of calcium, producing myofibrillar hypercontractility, mitochondrial damage, and myocardial stunning [22,24,25].

The accumulation of these ions produces an increase in intracellular osmolarity, which culminates in cellular swelling [22].

### 3.3. Nitric Oxide Metabolism

During episodes of ischemia-reperfusion, the nitric oxide synthase metabolic profile shifts and produces nitric superoxide [22]. Nitric oxide can also be produced through metabolic pathways via the xanthine oxidoreductase enzymatic complex and cytochrome C [23]. This molecule can produce oxidative damage by itself, or by serving as a substrate to produce reactive nitric oxide species [23].

Reactive nitric oxide species have the ability (similarly ro their reactive oxygen species counterparts) to produce damage to proteins, lipids, carbohydrates, and nucleic acids in tissue that has suffered ischemia-reperfusion injury [23].

### 3.4. Mitochondrial Dysfunction

Alterations in mitochondrial structure and function during ischemia-reperfusion injury diminish the amount of ATP available and produce the activation of metabolic pathways that result in cell death [25]. 

Ischemia enables the formation of the mitochondrial permeability transition pore, nevertheless, before perfusion is restored, this pore is closed because of the acid condition within the cell [23]. When the oxygen supply is restored, its sudden influx in combination with the accumulation of ROS and calcium ions promotes the pore’s opening; the result is the dissipation of the mitochondrial membrane potential, a reduction in the concentrations of ATP, and mitochondrial swelling [23,25]. Cytochrome C, a molecule that normally lies within the mitochondria and can activate the caspase system and therefore, induce apoptosis, can be found in the cell cytosol after the opening of the mitochondrial permeability transition pore [23].

### 3.5. Inflammation

Necrosis and cell damage induced by ischemia release multiple chemotactic molecules, such as TNF-α, IL-1, and platelet-activating factor, that act as recruiting signals for inflammatory cells [23]. 

Neutrophils and other leukocytes arrive at the affected region when perfusion is restored, their arrival to ischemic tissue is accompanied by the release of reactive oxygen species, reactive nitric oxide species, metalloproteinase, and hydrolytic enzymes, all of which produce injury to the parenchymal cells [23]. Furthermore, the infiltration of inflammatory cells activates tissue mast cells, which in return produces more damage to the tissue and further recruitment of leukocytes [23].

### 3.6. Cellular Death Pathways

Low levels of intracellular ATP activate cellular pathways such as serine/threonine kinases and protein kinase C, and these can induce autophagy, apoptosis, and necrosis [24]. The discussion of each of these pathways as separate entities is beyond the scope of this review. 

## 4. Experimental Studies

Numerous animal experiments have demonstrated the effectiveness of melatonin in reducing the severity of the myocardial ischemia-reperfusion injury.

Ischemia-reperfusion models in isolated rat hearts have shown that melatonin administration has a beneficial effect in reducing the severity of postischemic arrhythmias, limiting the area of infarction, protecting against myocardial dysfunction, and improving cardiac recovery [20,26,27,28,29].

Tan et al. conducted a study in which rat hearts were induced to 10 min of ischemia and then reperfused for 10 min; the percentage of the hearts that developed cardiac arrhythmias were recorded. The authors concluded that administration of melatonin (in doses of 1, 10, or 50 µM) either at the time of ischemia or at the time of reperfusion provides significant protection against ischemia-reperfusion arrhythmias [26].

Similarly, Lagneux et al. carried out an experimental study in which rat hearts were divided into 2 groups and were either put under regional ischemia for 5 min and then reperfused 30 min or put under 30 min of ischemia followed by 120 min of reperfusion. In the first group, pretreatment with melatonin significantly decreased the duration of reperfusion arrhythmias, while in the second group melatonin greatly limited infarct size. Therefore, it was found that melatonin and 5-MCA-NAT have similar protective functions when administered at doses of 10 mg/kg against myocardial ischemia-reperfusion injury [27].

Kaneko et al. studied rat hearts that underwent 30 min of ischemia followed by 30 min of reperfusion and determined that administration of melatonin before ischemia conferred protection against postischemic myocardial dysfunction and reperfusion arrhythmias. This way, melatonin decreases reperfusion arrythmia by decreasing lipid peroxidation and scavenging; the use of melatonin decreased ventricular tachycardia and ventricular fibrillation [28]. 

Likewise, Szárszoi conducted an experiment where rat hearts were exposed to 10, 15, or 25 min of ischemia and then reperfused for 10 min. It was found that perfusion hearts perfused with melatonin before ischemia protected against arrhythmias and contractile dysfunction associated with MI/R injury. Melatonin mainly reduced ventricular fibrillation and improved post-ischemic contractile function [29].

In a study carried out by Dobsak et al., rat hearts were subjected to 30 min of ischemia followed by 45 min of reperfusion, pretreatment with melatonin effectively reduced postischemic arrhythmias, protected the integrity of myocardium, limited the extent of lipid peroxidation, and suppressed apoptosis [20].

Sahna et al. conducted an experimental study in which an in vivo rat model went under ischemia for 30 min followed by a 120 min reperfusion. Results showed that melatonin (10 mg/kg) administered before ischemia was successful in reducing oxidative damage and infarct size (approximately 15%) [30].

In another study, Ekeløf et al. examined the therapeutic benefits of melatonin on the cardiovascular system in a porcine closed-chest infarction model. Ischemia was induced for 45 min followed by 4 h of reperfusion, however, distinct previous experimental studies, melatonin (200 mg) was administered just before reperfusion. Results showed that pretreatment with melatonin did not decrease myocardial reperfusion injury when evaluated by cardiac magnetic resonance. Therefore, the use of melatonin does not significantly increase the myocardial salvage index, nor does it significantly reduce the high-sensitive troponin T release [31].

Drobnik and colleagues demonstrated in rat experiments with coronary artery occlusion that physiological doses of melatonin (60 µg/100 g) for 28 days do not modify glycosaminoglycans, whereas administration of 60 µg/100 g melatonin increases them, but this dose does not modify collagen levels. Some rats were treated for 28 days with melatonin, while others were administered ethanol in 0.9% NaCl; after the 28th day, the rats were sacrificed to obtain the scars caused by the infarction [32]. In this experiment performed with in vitro cultures of mouse cardiac myofibroblasts obtained after coronary artery occlusion, pharmacological doses of melatonin were administered and compared with the effects of physiological concentrations. Exogen melatonin decreases GAG´s concentrations but does not modify collagen concentrations [32].

Physiological doses of melatonin produced an increase in collagen production depending on its plasma concentration; while in myocardial infarction there are 5–6 times more glycosaminoglycans in viable regions of the heart, using exogenous melatonin at doses of 300 µg/100 g decreases these concentrations, while collagen concentrations remain unchanged. In this experiment, glycosaminoglycans were studied after myocardial infarction because they are involved in the type of remodeling that the heart will undergo, for example, the excessive production of collagen causes heart stiffness. When the reperfusion ended, melatonin-treated hearts reached almost 93% of their pre-ischemic cardiac output values. The 30 min ischemia group developed fatal arrhythmias and fibrillation; after arterial occlusion melatonin doses between 0.5 to 5 mg/kg decreased premature ventricular contractions, ventricular tachycardia, and fibrillation [32,33,34].

In an experiment with isolated rat hearts subjected to an ischemia-reperfusion process, melatonin reduced the presence of postischemic arrhythmias, preventing infarct extension and improving postischemic cardiac recovery. Melatonin at doses of 50 and 100 mM effectively protects adult rat myocytes against reactive oxygen species (ROS) and intracellular Ca2 accumulation [20].

Experiments in mice have shown that melatonin promotes glucose metabolism and may prevent DM by promoting GLUT4 gene expression. Reduced serum melatonin levels have been associated with acute myocardial infarction and the formation of atherosclerotic plaques reducing the collateral blood flow. Impaired coronary microcirculatory responses following myocardial ischemia contribute to the aggravation hypertrophy and thereby lead to cardiac dysfunction [35]. Upon infarction the sarcolemma is damaged resulting in sarcoplasmic reticulum dysfunction, producing myocardial hypercontraction that leads to cell damage and apoptosis [36].

Hu et. al. conducted experiments with myocardial infarction models using Mst1 transgenic (Mst1 Tg) and Mst1 knockout (Mst1−/−) mice, in which pharmacological doses of 10 mg/kg/day of melatonin were administered for 3 weeks after induced ischemia. It was found that melatonin reduced the risk of cardiomyocyte apoptosis, presumably by increasing cardiomyocyte autophagy and alleviating mitochondrial dysfunction through the melatonin/Mst1/Sirt1 pathway. In this way, melatonin was found to alleviate intracellular stress by stabilizing mitochondrial dysfunction [37]. 

In another experiment performed by Singhanat et. al., neonatal rat cardiomyocytes were treated with melatonin after being exposed to a hypoxia-reoxygenation process. The pretreatment with melatonin administered 10 min before the ischemia-reperfusion process increased cell survival by reducing intracellular stress and thus the probability of apoptosis [38]. This process may induce autoimmune responses since reperfusion damage releases neoantigens that are recognized as damage-associated molecular patterns (DAMP’s), mainly by TLR 4 that induce the accumulation of immune system cells, this causes tissue damage by free radicals generation [39]. An overview of the previously exposed experimental studies is summarized in Table 1.

## 5. Clinical Studies

Seven clinical studies describing the effects of melatonin administration on myocardial ischemia/reperfusion (MI/R) and one systematic review and meta-analysis available at the moment are reviewed in this section.

The first study, conducted by Haghjooy and colleagues, studied the possible protective effect of melatonin on MI/R injury during coronary artery bypass graft (CABG) surgery by activating nuclear factor erythroid 2-related factor (Nrf2) as a key transcription factor used in the management of antioxidant defense at the cellular level, which was published in 2013. It was discovered that taking 10 mg of oral melatonin before bed for 1 month before surgery was linked with a substantial rise in both plasma levels of melatonin and Nrf2 concentration in the melatonin group compared to the placebo group in patients who underwent elective CABG. Due to melatonin’s beneficial effects in inducing activation of Nrf2, which can be used as a regulator guard sensitive of oxidative stress driving to the expression of important genes driven by antioxidant response elements, we concluded that it may play an important role in enhancing the antioxidant defense and reducing cell damage caused by CABG surgery through the Nrf2 pathway [43].

Ismail Gogenur and colleagues conducted further research in 2014 to see how perioperative melatonin therapy affected clinical cardiac morbidity and indicators of myocardial ischemia in patients who had elective surgery for an abdominal aortic aneurysm. The patients received injections of 50 mg melatonin or placebo intra-operatively and intravenously for 2 h, as well as 10 mg melatonin or placebo orally for the first 3 nights after the procedure. Blood samples were taken at 5 min, 6, 24, 48, 72, and 96 h following clamp removal/recirculation to assess postoperative cardiac morbidity. Holter monitoring was also used to continuously measure ST-segment depression. After abdominal aortic aneurysm repair, perioperative melatonin therapy reduced the incidence of clinical cardiac morbidity, troponin I levels, the frequency of ST-segment abnormalities, and the incidence of myocardial ischemia. With regards to the deviation of segment ST, no difference was found [44].

In 2015, Padideh Ghaeli and colleagues studied the effectiveness of melatonin in lowering cardiac biomarkers (highly sensitive troponin-T [hs-TnT] and creatine kinase-MB [CK-MB]) in patients undergoing percutaneous coronary intervention (PCI) which have ST-elevation myocardial infarction (STEMI). Patients were split into 2 groups: 1 received melatonin (3 mg orally delivered the night after PCI and continued every day during hospitalization) with conventional therapy, while the other received only conventional therapy. Preoperatively (baseline) and 6 h after the operation, Hs-TnT and CK-MB were measured. Melatonin substantially decreased CK-MB levels, although the average hs-TnT level did not change between the two groups. With this, it can be concluded that in the future after primary PCI melatonin might be considered as a routine regimen to reduce cardiovascular events [45]. The length of the research, the number of participants (30), and the low doses of melatonin were all cited as limiting factors by the authors.

Dwaich et al. investigated the protective effect of melatonin in reducing the severity of cardiac damage in patients undergoing CABG surgery, as well as whether this benefit was dosage dependent. From the fifth day before surgery, participants were randomly assigned to 1 of 3 research groups: placebo, treatment with melatonin at a low dose (10 mg/day), or treatment with melatonin at a high dose (20 mg/day). There was a substantial rise in ejection fraction in the melatonin groups, which was accompanied by a significant decrease in heart rate and a significant reduction in plasma levels of cardiac troponin-I, inducible nitric oxide synthase, interleukin-1β, and caspase-3 enzymes. When more carefully comparing the two melatonin groups, in the high dose melatonin group these changes were more significant. These data indicate that melatonin supplementation may reduce the severity of MI/R damage, by enhancing left ventricular (LV) function, which may be due to a dose-dependent effect mediated by decreasing oxidative stress, inflammation, and cardiac apoptosis [46].

In 2017, Dominguez-Rodriguez et al. investigated whether the timing of melatonin treatment in patients with STEMI affects its effectiveness. The authors analyzed post-hoc the Melatonin Adjunct in the Acute Myocardial Infarction Treated with Angioplasty (MARIA) study, which randomly assigned STEMI patients to melatonin (intravenous and intracoronary bolus) or placebo group during primary PCI. The interval from the beginning of symptoms and PCI was split into tertiles: first tertile (113–159 min), second tertile (177–215 min), and third tertile (208–290 min). Within one week of the primary PCI, magnetic resonance imaging was performed. The results showed that an intravenous bolus of 51.7 μmol melatonin was given 60 min before reperfusion and an intracoronary bolus of 8.6 μmol melatonin was given at the beginning of reperfusion reduced infarct size (total 14 mg) but did not enhance LV function. When melatonin is given <2.5 h after the beginning of chest pain, according to cardiovascular magnetic resonance imaging measurements, the infarct size reduced by about 40%. In comparison to delayed melatonin treatment, the authors concluded that early administration of melatonin may have stronger cardioprotective benefits [47].

Sarah Ekeloef et al. proposed in 2017 that melatonin administration during acute cardiac reperfusion increases myocardial salvage index (which is a measure of treatment efficacy). At the start of reperfusion, patients were given either 0.1 mg/mL melatonin intracoronary and 0.1 mg/mL melatonin intravenous (total 50 mg) or a placebo. The myocardial salvage index (evaluated by cardiac magnetic resonance on the 4th day [±1 day] after primary PCI) was almost identical in the melatonin and placebo groups (55.3 percent and 61.5 percent, respectively), indicating that melatonin did not affect LV function or infarct size. Both groups had almost equal amounts of hs-TnT, CK-MB, and oxidative biomarkers. At 90 days, there was no difference in the occurrence of major clinical events between the 2 groups. Finally, as compared to placebo, melatonin did not enhance myocardial salvage index after primary PCI in patients with STEMI [48].

Ebrahim Shafiei et al. evaluated the effectiveness of oral N-acetylcysteine (NAC) and melatonin intake in decreasing early reperfusion injury and acute oxidative stress in CABG patients in 2018. The researchers measured the levels of lactate, cardiac troponin I, tumor necrosis factor-alpha (TNFα), and malondialdehyde (MDA) in the blood. A total of 88 patients were categorized into 3 groups; the first group received a placebo, the second group received melatonin tablets of 5 mg (3 times beginning from 24 h before the procedure and a single dose [15 mg] 1 h before the surgery) and the third group got NAC (600 mg orally 3 times a day starting 2 days before the procedure, and a single dose in the morning of the CABG surgery). Blood samples were collected at 6 different times: preoperatively, before aortic clipping, during aortic clipping, before the commencement of reperfusion, 15 min after reperfusion, and finally after recovery in the intensive care unit. Lactate, TNF-α, MDA, and troponin I levels were observed to be lower in the melatonin and NAC groups. MDA, lactate, TNF-α, and troponin I had statistically significant differences in average serum levels between the melatonin and control groups (*p* < 0.001, *p* = 0.001, and *p* = 0.001, respectively). The sole difference between the control groups and NAC, as well as the NAC and melatonin groups, was the average lactate level (*p* < 0.001). It was discovered a significant difference in the mean value of total intensive care unit and hospital durations between the control and melatonin and NAC groups as a clinical result. Melatonin and NAC were found to prevent CABG-related cardiac damage [49].

In 2021, Dominguez-Rodriguez et al. conducted a systematic review and meta-analysis of the existing literature. Multiple randomized control studies that utilized melatonin to decrease the size of infarcts were found. In individuals who received myocardial revascularization and were randomized to receive melatonin or placebo, the main outcomes were: left ventricular ejection fraction (LVEF) and blood troponin levels. A total of 7 randomized controlled trials (RCTs) fulfilled all of the inclusion criteria out of a total of 283 records examined. The patients treated with melatonin exhibited a greater LVEF on average than the ones who received a placebo. Troponin I levels were also lower in the patients’ receiving melatonin. Melatonin treatment in humans reduced cardiac dysfunction and improved LVEF and troponin levels, according to the authors [50].

The variations in the severity of acute myocardial infarction (AMI), the timing of melatonin delivery, and the age of the patients in each trial might explain the seemingly disparate outcomes. In comparison to late melatonin treatment, early melatonin administration may lead to stronger cardioprotective benefits [38]. Furthermore, they have also seen that the routes of melatonin delivery may have influenced the results, being better orally. Orally given melatonin and its metabolites may have better pharmacokinetics than other approaches [39]. The fact that around 30% of patients with MI/R injury have hyperlipidemia, 40% have diabetes and 50% have hypertension must also be considered [51]. These have a role in the progression of MI/R damage and make treatment more difficult [52].

In the majority of these trials, it can be inferred that melatonin therapy improves LVEF or reduces infarcted area as compared to placebo, but it has to be taken into consideration that this depends on the route of administration, timing and dosage. Moreover, almost all of the melatonin-treated groups had a substantial drop in troponin plasma levels. When melatonin was administered orally, the findings on LVEF were homogeneous across all trials, however, there was substantial variability in the degree that CK-MB or troponin levels dropped. Nonetheless, lower CK-MB or troponin levels in individuals receiving melatonin was found across all the studies, so the only discrepancy refers to the amount of troponin levels that this medication can decrease [50]. The above-mentioned studies are summarized in Table 2.

All of the exposed studies used a greater number of men than women, both in the control groups and in those that were administered melatonin. None of them compared the outcomes between the genders to see if there were any differences. It is well-known that differences in women with myocardial infarction have a close relationship with sex hormones, therefore it would be interesting to open more lines of research on this topic.

## 6. Melatonin Analogs

As discussed above, the potential therapeutic use of melatonin is limited due to its poor half-life and low bioavailability. DeMuro and colleagues report that only 15% of oral ingested melatonin reaches the bloodstream, which may be due to decreased oral absorption in combination with increased first-pass metabolism in the liver. Therefore, doses of melatonin commonly used for pharmacological treatment have very poor absorption. Moreover, since there are no pharmacodynamic relationships for the different therapeutic purposes of melatonin, this low bioavailability may not be sufficient to cover the minimum drug exposure required, depending on the indications for which it is prescribed [53]. 

However, due to the therapeutic potential of this molecule, in the past few years, efforts have been centered on the research and development of melatonin analogs with improved pharmacokinetics and that display higher receptor affinity with melatonin receptors [54] (Table 3).

Ramelteon, agomelatine, and tasimelteon are the melatonergic drugs currently approved for the treatment of sleep and circadian disturbances [55]. While other drugs under development are piromelatine (Neu-P11) and TIK-301 [56].

Since these compounds are aimed at treating conditions related to circadian dysfunction, and there is very limited clinical information on whether melatonin analogs produce any effect on the cardiovascular system, it is difficult to predict if they also share the cardioprotective effects of melatonin. Furthermore, it appears that these melatonergic drugs do not share the antioxidant effect of melatonin, except for 5-MCA-NAT, a structural melatonin analog that is selective for the MT3 receptor, inhibiting its quinone reductase action and conferring antioxidant properties [57,58].

Interestingly, regarding 5-MCA-NAT, Lagneux et al. conducted a study in an isolated rat heart model to determine if melatonin and 5-MCA-NAT were able to protect against the incidence of arrhythmias and decrease the infarct size resulting from ischemia-reperfusion injury. Results showed that although both compounds were effective in reducing arrhythmias and infarct area, the beneficial effect of 5-MCA-NAT was superior to that of melatonin [28].

The majority of research trials examining melatonin’s cardioprotective therapeutic function are now in phase 2a. The selective melatonin receptor agonists tasimelteon, ramelteon, and mixed melatonergic-serotonin agomelatine, as well as additional agonists with structures recorded in ChEMBL (a database of bioactive molecules), have not yet been studied in humans as cardioprotective medicines. Melatonin receptor agonists are superior to melatonin in terms of having more well-defined pharmacologic characteristics and comprehensive safety data. The structure of the MT1 and MT2 receptors (as determined by X-ray crystallography) should help us for the creation of better highly selective melatonin receptor agonists. Melatonin appears to have a cardioprotective effect in a variety of cardiac disorders, according to preclinical proof-of-concept and early clinical trials (phase 2a) [59]. The available data of 4 of these agonists is exposed.

In 2018, Martin Stroethoff and colleagues studied the possible reduction in infarct size through a postconditioning mechanism by using ramelteon (a therapeutically utilized insomnia medication that works by activating melatonin receptors). Six sets of male Wistar rats were created. Each group went through 3 periods: an initial baseline of 20 min baseline, ischemia of 33 min, and reperfusion of 60 min. The different study groups experienced the following protocols after the ischemia period and at the start of reperfusion: treatment of the heart with melatonin and ramelteon. Luzindole (melatonin antagonist), was given with and without melatonin and ramelteon, respectively. The authors waited for the reperfusion, and then the heart was cut into transverse slices and dyed with a 0.75% triphenyltetrazolium chloride solution. Infarct area size was determined by area measurement method using SigmaScan Pro 5 computer software. It was firstly observed that ramelteon reduces infarct size to the same degree as melatonin, and secondly that luzindole fully eliminates these effects. Something important to take into consideration is that ramelteon itself does not have antioxidant properties, such as melatonin or luzindole, which means that the cardioprotective effect of ramelteon is probably only mediated by the melatonin receptor activation, as well as the activation of the mitochondrial calcium-sensitive potassium channels and the activation of the mitochondrial adenosine triphosphate-sensitive channels. Their findings showed that ramelteon can decrease MI/R injury of isolated rat hearts by approximately 50%. Ramelteon similarly reduced infarct size as melatonin. Since ramelteon is a medication with selectivity for MT1 and MT2 receptors and a favorable side effect profile [60], this discovery may be of special therapeutic significance. There have been no clinical studies of ramelteon’s effectiveness in the treatment of cardiac or cardiovascular disorders to date.

In 2014, Jianwu Yu and colleagues investigated the mechanism of piromelatine (nonselective melatonin receptor agonist) in cardiac cells damaged by hypoxia/reoxygenation damage. H9c2 cardiac cells were used to create a hypoxia/reoxygenation model, and the cells were split into three groups: control, hypoxia/reoxygenation, and piromelatine. Levels of creatinine kinase, lactic dehydrogenase, superoxide dismutase, and malondialdehyde in cell culture medium, as well as the apoptosis rates of cardiac cells were compared in various groups.

Compared with the hypoxia/reoxygenation group, the levels of creatinine kinase, lactate dehydrogenase, and malondialdehyde in the piromelatine group were substantially lower, although both were significantly higher than those in the control group. In addition, the amount of superoxide dismutase was raised while malondialdehyde was lowered in the cells, which leads to a decrease in lipid peroxidation and protects the mitochondria from MI/R injury. It was determined that piromelatine protects cardiac cells from hypoxia-reoxygenation damage. It reduces lipid peroxidation and protects mitochondria from myocardial ischemia/reperfusion injury, it inhibits cell apoptosis and improves the rhythm and morphology of cardiac cells; it also reduces cell membrane damage by decreasing cell membrane permeability, which might stabilize myocardial cell membrane [61]. There have been no clinical studies of piromelatine’s effectiveness in the treatment of cardiac or cardiovascular disorders so far.

In 2018, Pengyu Jia and colleagues used an isolated rat heart model to investigate the effects of agomelatine (a melatonin [MT1/MT2] receptor agonist and serotonin [5-HT2C] receptor antagonist) on MI/R damage. To achieve MI/R injury, first, the rat hearts were isolated and submitted for 30 min to ischemia, then waited 120 min for it to be reperfused. Furthermore, 1 h before separating the heart, rats were given an intraperitoneal injection of agomelatine (10, 20, or 40 mg/kg). Agomelatine (20 mg/kg and 40 mg/kg) significantly improved cardiac function, alleviated pathological changes in the ischemic myocardium, reduced myocardial infarct size, and decreased release of CK-MB and lactate dehydrogenase, inhibited mitochondrial permeability transition pore opening. Notably, agomelatine’s protective effects were abrogated by atractyloside, a mitochondrial permeability transition pore opener. It was also found that agomelatine significantly enhanced GSK-3β phosphorylation and decreased the expression of cytochrome C, cleaved caspase 9, and cleaved caspase 3, resulting in a decreased apoptosis rate. Agomelatine (20 mg/kg and 40 mg/kg) improves heart function, decreases pathological alterations in ischemic myocardium, reduces the area of myocardial infarction, slows the opening of mitochondrial transport pores, and reduces the release of CKMB and lactate dehydrogenase. Atractyloside, in particular, eliminates agomelatine’s protective action (a transitional pore opener for mitochondrial permeability). The authors also discovered that agomelatine increased GSK-3β phosphorylation while lowering the production of cytochrome C, cleaved caspase 9, and cleaved caspase 3, resulting in a lower apoptosis rate. These data suggest that agomelatine protects against MI/R damage by blocking the opening of mitochondrial permeability transition pores [62].

Just one melatonin analog, the tasimelteon, has not been yet investigated in the context of heart or cardiovascular disorders, which is promising since it is a selective dual MT1 and MT2 receptor agonist [59].

There are a few contraindications for using melatonin analogs such as renal failure, liver failure, high lipid levels, and alcohol addiction. The adverse effects that they can cause, although few, include headache, rebound insomnia, nausea, elevations in liver parameters; when used for 6 to 12 months, melatonin can also cause withdrawal symptoms or even addiction [63].

Although there is a large amount of preclinical evidence that supports the use of melatonin for cardiovascular protection, a better understanding of the pharmacokinetics and pharmacodynamics of melatonin and its related analogs will help to integrate the results of basic research into clinical practice [59]. This is significant since the role of melatonin and its agonists as cardioprotective medical agents require bigger phase 3 randomized intervention trials. Melatonin receptor agonists are superior to melatonin in terms of better pharmacological characteristics and more selective effects [59].

As a result, given experimental and clinical studies confirm melatonin’s positive impact on the cardiovascular system, melatonin analogs may potentially provide cardiovascular protection. This assertion, however, requires additional experimental and clinical data to be confirmed. This might be a crucial field of study, especially given the increasing prevalence of cardiovascular illnesses across the world [56].

## 7. Conclusions

Melatonin is a promising molecule that confers a cardioprotective role in ischemia-reperfusion injury, it has a good safety profile, and it could be widely used and distributed in the clinical settings. Despite the multiple experimental and clinical studies that support its use, further studies with larger populations are needed to recommend melatonin as part of the standard treatment in myocardial ischemia. To date, available studies have demonstrated that variables such as left ventricular ejection fraction and presence of cardiac arrhythmias are the main conditions that benefit more from melatonin administration. Nevertheless, future trials should evaluate clinical outcomes as well, such as mortality, functional class improvement, presence of symptoms—such as angina—and perhaps length-of-stay for patients treated with this agent. Melatonin analogs appears to possess the antioxidative and anti-inflammatory properties of melatonin with the added benefit of their pharmacokinetics, however, there is a lack of clinical studies evaluating their safety and efficacy in humans. In the future, melatonin—or even perhaps melatonin analogues—could be incorporated in the standard treatment recommended by international societies for the management of myocardial infarction, until then further research is needed and in the case of melatonin analogues, the optimal administration route will have to be elucidated and hopefully the ischemia-reperfusion injury could be set aside in few years. 

## Figures and Tables

**Table 1:** Experimental studies that assessed melatonin’s efficacy for ischemia-reperfusion injury.

Experimental Studies				
Experiment	Animals or Tissues Used	Melatonin Dosage	Outcome	Major Findings
After melatonin pretreatment, rats hearts were excised and perfused retrogradely using the Langendorff technique, then ischemia was induced by left coronary artery ligation [28]	Male Wistar rat hearts	10 mg/kg	Pretreatment with melatonin conferred protection against arrhythmias caused by the infarction, as well as reduced the damaged area	Melatonin and 5-MCA-NAT have similar protective functions when administered at doses of 10 mg/kg against myocardial ischemia-reperfusion injury
Rats hearts were excised and perfused retrogradely using the Langendorff technique, then ischemia was induced followed by 30 min of reperfusion with melatonin [29]	Male Sprague-Dawley rats	100 µmol	Melatonin had a protective effect against myocardial reperfusion injury	Melatonin decreases reperfusion arrhythmia by decreasing lipid peroxidation and scavenging. The use of melatonin decreased ventricular tachycardia and ventricular fibrillation
Regional ischemia induced by left anterior descending coronary artery occlusion [30]	Adult male Wistar rats	10 µmol/L	Melatonin decreased the incidence and severity of ventricular arrhythmias	Melatonin mainly reduced ventricular fibrillation and improved post-ischemic contractile function
Rat hearts were connected to perfusion cannulas of Langendorff apparatus, this cannula will be closed for the ischemia process for 30 min and then opened for reperfusion for 45 min [16]	Male Wistar rats	50 and 100 mM of melatonin in KHB solution	Melatonin reduced dangerous oxidation	Melatonin does not have an oxidation-reduction cycle and therefore prevents oxidative damage to cellular components
Mouse coronary artery occlusion (Left coronary artery ligation) [33]	In vitro cultures of mouse cardiac myofibroblasts	300 µg/100 g	Melatonin modified cardiac remodeling	Exogen melatonin decreases GAG´s concentrations but does not modify collagen concentrations
Myocardial infarction/reperfusion surgery [40]	Male Sprague Dawley rats	10 mg/kg/day for 4 weeks	Prophylactic use of melatonin improved cardiac function	Melatonin reduces apoptosis by reducing caspase 3 expression when Notch1/Hes1 signaling is stimulated
Myocardial ischemia/reperfusion (MI/R) induced injury exacerbated by CIH [41]	Adult male Sprague Dawley rats	10 mg/kg	Melatonin reduced myocardial inflammation, fibrosis and exacerbated MI/R injury	Systolic pressure, heart weights, and malondialdehyde were significantly increased in hypoxic rats but not in the melatonin-treated group Rats treated with melatonin have reduced levels of inflammatory cytokines (TNF-α, IL-6, and COX-2) and fibrotic markers (PC1 and TGF-β)
Chronic intermittent hypoxia [42]	Rats	10 mg/kg melatonin or saline solution daily for six weeks	Melatonin reversed myocardial hypertrophy	The activation of AMPK signaling activates autophagy reducing myocardium apoptosis
Closed-chest porcine model of myocardial ischemia and reperfusion [32]	Female Danish Landrace pigs	200 mg (0.4 mg/mL)	The combination of intravenous and intracoronary melatonin did not reduce myocardial reperfusion injury.	The use of melatonin does not significantly increase the myocardial salvage index, nor does it significantly reduce the high-sensitive troponin T release
MI model using Mst1 transgenic (Mst1 Tg) and Mst1 knockout (Mst1−/−) mice [37]	Mice	10 mg/kg/day of melatonin for 3 weeks	Melatonin reduced cardiomyocyte apoptosis	Melatonin was found to alleviate intracellular stress by stabilizing mitochondrial dysfunction
Hypoxia/reoxygenation process [38]	Cardiomyocytes from neonatal rats and H9C2 cells	-	Melatonin activated some signaling pathways that protect cardiomyocytes from oxidative stress	Melatonin protects heart cells by activating survival via JAK/STAT, STAT3, Notch1/Hes1, PKG1α, PI3K/Akt, ERK1/2, and AMPKα reducing mitochondrial and cellular oxidative stress, mitochondrial fission, endoplasmic reticulum stress, and apoptosis

**Table 2:** Clinical studies on melatonin’s efficacy in ischemia-reperfusion injury. Coronary artery bypass graft (CABG), acute myocardial infarction, (AMI), nuclear factor erythroid 2-related factor (Nrf2), ST-elevation myocardial infarction (STEMI), percutaneous coronary intervention (PCI), creatine kinase-MB (CK-MB), high sensitive troponin-T (hs-TnT), left ventricular ejection fraction (LVEF), heart ratio (HR), cardiac troponin I (cTn-I), myocardial ischemia/reperfusion (MI/R), left ventricular end-diastolic volume (LVEDV), left ventricular end-systolic volume (LVESV), left ventricle (LV), malondialdehyde (MDA), tumor necrosis factor alpha (TNF-α), N-acetyl cysteine (NAC).

Clinical Studies							
Study Model	Sample Size	Melatonin Administration	Reperfusion Medical Strategies	Major Findings			Interpretation
				Lv Function/Hemodynamic Parameters	Infarct Size	Biomarkers	
Elective CABG None with AMI [39].	30	Before bedtime, orally 10 mg of melatonin 1 month before the procedure	CABG	-	-	↑ Melatonin ↑ Nrf2	Melatonin, through the Nrf2 pathway, may have a key role in the potentiation of antioxidant defense and mitigation of cellular damages caused by CABG surgery.
Elective surgery for abdominal aortic aneurysm [43].	50	Intraoperatively and intravenously 50 mg melatonin over 2 h; and orally 10 mg of melatonin throughout the first 3 nights after the procedure	CABG	-	-	↓ Troponin-I	Clinical cardiac morbidity, troponin I levels, the frequency of ST-segment deviations, and the incidence of myocardial ischemia were reduced following the procedure.
Patients with STEMI [44].	40	The night following PCI melatonin 3 mg was orally given and maintained daily in the hospital	PCI	-	-	↓ CK-MB 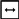 hs-TnT	There is no impact. The length of the trial, the sample size, and the low doses of melatonin used were all limitations.
Ischemic heart disease patients undergoing elective CABG [45].	45	From the fifth day before surgery, a low dosage melatonin therapy group (10 mg/day) and a high dosage melatonin treatment group (20 mg/day) were used	CABG	↑ LVEF ↓ HR	-	↓ cTn-I ↓ Interleukin-1β ↓ Inducible nitric oxide synthase ↓ Caspase-3 enzymes.	Melatonin reduced oxidative stress, inflammation, and apoptosis in ischemic heart disease patients following CABG, decreasing MI/R injury.
ST-elevation myocardial infarction patients [46].	146	An intravenous bolus of 51.7 μmol melatonin was given 60 min before reperfusion, followed by an intracoronary bolus of 8.6 μmol (total 14 mg) melatonin at the start of reperfusion.	Primary PCI	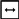 LVEDV 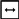 LVESV 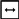 Total LV mass	↓ Infarct size with symptoms starting 136 ± 23 min later 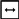 Infarct size with symptoms starting between 196 ± 19 min and 249 ± 41 min later	-	In STEMI individuals, early melatonin treatment decreased infarct size.
ST-elevation myocardial infarction patients [47].	48	0.1 mg/mL melatonin intracoronary and 0.1 mg/mL melatonin intravenous injection (total 50 mg)	Primary PCI	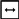 LVEDV 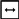 LVESV 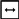 LVEF	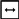 Infarct size	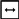 hs-TnT, CK-MB	Melatonin did not affect LV function or clinical outcomes in STEMI patients.
Elective CABG [48].	88	Melatonin 5 mg given orally (3 times beginning from 24 h before the procedure and a single dose [15 mg] 1 h before the surgery)	CABG	-	-	↓ Troponin I ↓ Lactate ↓ MDA ↓ TNF-α	NAC and melatonin are powerful antioxidants that have almost equal effectiveness in decreasing CABG-related heart damage and oxidative stress at the doses used in the study.

**Table 3:** Experimental studies that assessed melatonin’s analogs efficacy for improving the effects of ischemia-reperfusion injury.

Experimental Studies of Melatonin Analogs				
Melatonin Analog	Animals or Tissues Used	Conduct of the Experiment	Outcome	Major Findings
5-MCA-NAT [27]	Wistar rats	Rats were treated with either melatonin or 5-MCA-NAT (10 mg/kg) and divided into two groups: regional ischemia for 5 min and reperfused for 30 min, and regional ischemia for 30 min and reperfused for 120 min	Pretreatment with either melatonin or 5-MCA-NAT conferred protection against arryhtmias caused by the infarction and reduced the infarction size	5-MCA-NAT shows a significant protection against ischemia-reperfusion injury. These protection is very similar to that of melatonin
Ramelteon [60]	Male Wistar rats	Six sets of rats that underwent ischemia-reperfusion: Treatment with melatonin and ramelteon, treatment with melatonin and luzindazole, and treatment with ramelteon and luzindazole	Ramelteon reduces infarct size to the same degree as melatonin and that luzindazole fully eliminates the effects od ramelteon	Ramelteon can decrease MI/R injury by approximately 50%, and because this compound has selectivity for MT1 and MT2 receptors and a favorable side effect profile, it may be of special therapeutic significance
Piromelatine [61]	H9c2 cardiac cells	H9c2 cardiac cells were used to create a hypoxia/reoxigenation model and cells were split into three: control, hypoxia/reoxigenation and Piromelatine. CK, LDH and SOD were compared	CK, LDH and MDA were lower, and SOD was raised in the Piromelatine group	Piromelatine protects cardaic cells from hypoxia-reoxigenation damage. It reduces lipid peroxidation and protects the mitochondria from MI/R injury, inhibits apoptosis, improves rhytm and morphology of cardiac cells, and reduces membrane permeability
Agomelatine [62]	Rats	Rat hearts isolated and and submitted to 30 min of ischemia. After 120 min, they were reperfused. Rats were given an intraperitoneal injection of 10, 20 or 40 mg/kg of agomelatine	Agomelatine improved cardiac function, alleviated changes in ischemic myocardium, reduced infarct size, decreased CK-MB and LDH, inhibited de MPTP. Agomelatine also decreased cytochrome C, caspases and enhaced GSK-3β phosphorylation	The data suggest that agomelatine protects against MI/R by blocking the opening of mitochondrial permeability transition pores
